# Supplementary material for: Characterization of hemodialysis fistulas experienced abrupt thrombosis and determination of a proper follow-up protocol: A retrospective cohort study and an interventionist’s perspective
Source: PLoS One. 2023 Mar 13;18(3):e0282891. doi: 10.1371/journal.pone.0282891 (PMC10010559; doi:10.1371/journal.pone.0282891)
Supplement: Attachment — Submitted filename: 27.1.2023.docx [file pone.0282891.s001.docx]

The present manuscript has potentially important data on an interesting topic how to get better patency for failing artery - venous fistula. The cohort is reasonable sized 227 patients. The main issue with the manuscript is that it would have been easier to review if authors had finalized the manuscript little more. The order of presented issues could be more logical, results and observations should be more accurately presented, tables should have all appendixes explained and legends be more detailed and even figure legends were not on a file for the review.

Also it would be interesting to have a demographic data correlated with the AVF requiring additional procedures and the ones that function just fine.

Abstract: Some parts of the abstract are hard to follow and AVF both with and without thrombosis should be clearly defined when presenting results.

Introduction: There should be a strong body of literature on AVF thrombosis and patency, relevant and as scientific base for the introduction. At present introduction lacks the scientific touch.

Methods: Sample characteristics with the statement of ethical approval details etc. would define the work better? The methods could be better formulated to be easier to read and interpret. Also, some of the nice flow charts could be in supplement? Essentially at present methods is hard to follow and still does not include required details.

Results: Over 500 VA and only little over 227 enrolled rationales? Figure 3 shows the explanation, but authors should either refer to figure 3 after stating 227 enrolled or explain briefly why half of the VA were excluded. It would be nice to have more detailed demographic data presented.

Tables should have subheading containing the appendixes written out and be more comprehensive.

Figures should have detailed legend? Now these legends seem to be missing.

The discussion should be more fluent.
